# Abortive Treatment of Whooping-Cough with Sulph. Quinine

**Published:** 1876-11

**Authors:** 


Abortive Treatment of Whooping-cough with Sulph. Quinine.—Dr. Rufus K. Hunton, of Philadelphia, reports to the *Medical and Surgical Reporter* ten cases of whooping-cough cured by quinine, as follows:

"Case 1.—I was called to see John W., the 10th of November last, with whooping-cough complicated with pneumonia; found the patient suffering with great prostration, difficult breathing; pulse 120 and feeble; temperature under the tongue 103; harassed with a hacking cough, with a whooping coming on every hour, which lasted for some time, and completely prostrated the patient. To sustain the patient's strength, I placed him on small doses of brandy every half hour, with milk punch made with sherry wine, and beef tea; and, for its antipyretic effect, ordered quinine in three-grain doses every three hours, with turpentine stupes to top of right lung, and hot poultices of wheat bran over both lungs. 11th. On visiting the patient next morning, found a decided improvement; the skin moist; temperature 100; pulse 100, with a strong, full beat, the whoop coming on every three or four hours. Continued treatment, leaving off the turpentine stupes. 12th. The patient much improved; temperature 99; pulse 90; the whooping, coming on every four or five hours, was of short duration. The same treatment continued. 13th. The boy says he is so much better he would like to sit up in bed; whooped three times since my visit yesterday; temperature 98½; pulse 85. Treatment continued. 14th. Whooped only one time since yesterday; temperature 98¼; pulse 80. The patient continued to improve, the whoop disappeared on the fifth day, and by the tenth day he was out of bed. Attributing the cessation of the whoop to the quinine, I resolved to try its effect in aborting whooping-cough.

Cases 2 and 3 occurred in my own family in December last, and as the patients were both going to school, and their examination was coming off the 1st of January, and any loss of time would prevent their promotion, I placed them on quinine, in five-grain doses, in the morning before going to school, five grains at three o'clock in the afternoon when returning from school, five grains at bedtime, given in wafers. Both cases were slight, without complications; the whoop disappeared in one on the third day, in the other on the second day; both went to school without losing a day.

Case 4.—Joseph R., boy, four years old. Called to see this patient the 5th of January. Complicated with a slight bronchitis, hacking cough, whoop coming on every three hours. Ordered quinine, in two-grain doses, every three hours, in wafers, with a mixture containing muriate of ammonia, syrup tolu, and an anodyne, to quiet bronchial cough, with hot poultices to chest. On the fourth day the whoop was entirely gone, leaving a slight bronchial cough, which was well by the sixth day.

Cases 5 and 6.—Annie W., girl, ten years old; Robert W., boy, eight years old. Was called to see both cases on the 20th of February last; both slight cases, with no complications. Ordered quinine, in two-grain doses, every three hours. In the girl the whoop was aborted in twenty-four hours, in the boy in thirty hours.

Case 7.—Patrick O., teamster, twenty-four years old, called at my office last February; stated he thought he had whooping-cough, and while in the office a paroxysm of coughing came on, with a distinct whoop, which confirmed the diagnosis of the disease. Ordered quinine, three-grain doses, to be made in pills, every four hours. Told him to call at my office on the third day. He called on the morning of the fourth day, reporting himself cured, as he had not whooped since he had taken the seventh pill; did not stop his work.

Case 8.—Maggie F., girl, eighteen months. Was called to see her the sixth of January last; mild attack. She was ordered quinine, in one-grain doses, every three hours. In thirty-six hours after taking the first dose the whoop ceased.

Cases 9 and 10.—On the 10th of March last, I was called to see Katie and Pauline S., girls, one five years old and the other seven. I placed both patients on the old remedies—alum, belladonna, bromide ammonium, nitric acid. The disease ran for four weeks without any abatement of the cough. Believing that the disease would run for some time with the whoop, which had already confined the patients to the house, I ordered for both quinine sulph., in two-grain doses, every three hours. In one case the whoop was aborted on the second day; in the other case the whoop was cut short in three days.

To avoid the nauseous, bitter taste of the quinine, which is so repugnant to all patients, especially children, that it is exceedingly difficult to get them to take the medicine, and when taken they almost invariably eject it from the stomach, I order the powders of quinine put up in cachets, or wafers, and by dipping the wafer in water, so as to soften it, and placing the moistened wafer in a spoon filled with water, and placing the spoon with the water on the back part of the tongue the smallest child can swallow the wafer and retain the quinine without vomiting."

